# Multimodal ultrasonography for predicting epidermal growth factor receptor mutation in subpleural non-small cell lung carcinoma

**DOI:** 10.3389/fonc.2025.1722238

**Published:** 2026-01-07

**Authors:** Jing Bai, Qifei Zhang, Song Wang, Hong Wang, Kun Yan, Wei Zhou, Liang Dong, Wei Yang

**Affiliations:** 1 Key Laboratory of Carcinogenesis and Translational Research (Ministry of Education/Beijing), Department of Ultrasound, Peking University Cancer Hospital & Institute, Beijing, China; 2 Department of Ultrasonography, Shengli Oil Field Central Hospital, Dongying, Shandong, China

**Keywords:** contrast-enhanced ultrasound, EGFR mutation, non-small cell lung cancer, predictive model, ultrasonography

## Abstract

**Objective:**

Currently precise target treatment based on gene status significantly improved the outcome for patients with non-small cell lung cancer (NSCLC) and epidermal growth factor receptor (EGFR) was the most important gene. We aimed to develop a multimodal ultrasound model for predicting EGFR mutation status in patients with subpleural NSCLC, to provide important information for precise target treatment.

**Methods:**

75 patients with pathologically confirmed NSCLC were included in this retrospective study. Patients were divided into two groups based on EGFR mutation status: wild-type (n=57) and mutant (n=18). The clinical characteristics (C), conventional ultrasound (US) features, contrast-enhanced ultrasound (CEUS) characteristics, and time-intensity curve (TIC) parameters of the lung lesions were analyzed and compared between the two groups. Univariate and multivariate logistic regression determined independent predictors of EGFR mutations. Two predictive models were constructed: a C+ US model and a FULL model. Both were presented using nomograms. Receiver operating characteristic and calibration curves evaluated predictive performance of two models, while decision curve analysis (DCA) assessed clinical utility.

**Results:**

Multivariate analysis identified smoking status, lesion boundaries, and air bronchogram as predictors in the C + US model. The FULL model identified lesion boundaries and air bronchogram on US, enhancement intensity of lesions and internal necrosis on CEUS and RT (rise time) from TIC as predictors. The C + US model achieved an AUC of 0.843, and the FULL model achieved 0.939. DCA confirmed substantial net clinical benefits.

**Conclusion:**

The models developed in this study enabled patients who are unable to undergo invasive procedures to predict EGFR mutation status noninvasively. These findings provided an ultrasound-based diagnostic reference to support clinician decision-making and personalized treatment planning.

## Introduction

Lung cancer persists as the predominant contributor to global cancer-related morbidity and mortality, with non-small cell lung cancer (NSCLC) constituting approximately 85% of cases (1). Recent advances in molecular biology have identified several driver mutations, including epidermal growth factor receptor (EGFR), BRAF, ROS1, MET, and ALK, which are closely related to the pathogenesis and progression of lung cancer (2). Consequently, targeted therapies have transformed the treatment landscape for individuals with NSCLC (3, 4). Notably, epidermal growth factor receptor tyrosine kinase inhibitors (EGFR-TKIs) are widely administered to individuals harboring EGFR mutations and have been shown to significantly prolong survival (5, 6). The decision to initiate targeted therapy relies on EGFR mutation status evaluation (7) which achieved through pathological examination. However, this process can be limited by the general condition of the patients, biopsy operation ability of the doctors, sample quality and intratumoral heterogeneity (8–11). Therefore, the development of noninvasive methods to predict EGFR mutation status is essential to complement gene sequencing.

Although CT remains essential in lung cancer imaging, it involves exposure to ionizing radiation (12). In contrast, multimodal ultrasound imaging offers high sensitivity, real-time monitor, and nonionizing advantages in evaluating peripheral lung lesions. Its utility in distinguishing benign from malignant pulmonary lesions and in identifying histological subtypes of lung cancer has been well-proven by our center and other studies (13, 14). Beside the role of characterization of lung lesions with CEUS, we further explored the perspective of CEUS in prediction of gene status which related to target therapy. In this study, we aimed to identify ultrasound (US) and contrast-enhanced ultrasound (CEUS) features associated with EGFR mutation status and to construct predictive models for assessing EGFR genotype. To our knowledge, this work was the first study to apply ultrasonographic imaging for predicting EGFR mutation status in subpleural non-small cell lung cancer.

## Materials and methods

### Study design and patients

**Figure 1:**
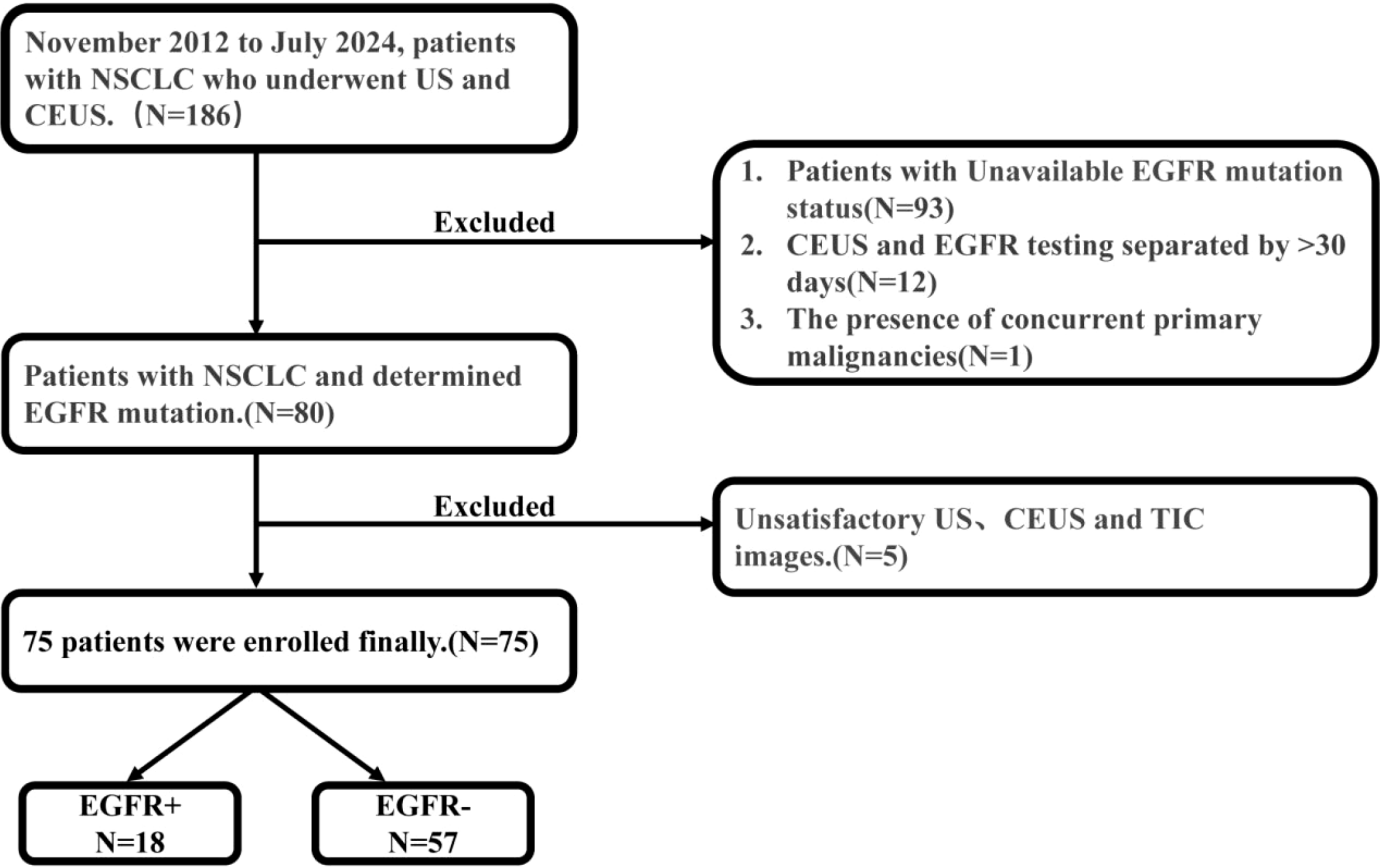
Flow diagram of the patient’s selection. EGFR, Epidermal Growth Factor Receptor; US, ultrasound; CEUS, contrast enhanced ultrasound; NSCLC, non-small cell lung cancer; TIC, time-intensity curve.

From November 2012 to July 2024, totally 186 consecutive patients with confirmed peripheral lung NSCLC underwent US and CEUS examination at our department were collected retrospectively. The inclusion criteria were as follows: (i) age ≥18 years; (ii) histologically confirmed NSCLC; (iii) documented pretreatment EGFR mutation status; and (iv) availability of pretreatment US and CEUS images. Exclusion criteria were as follows: (i) an interval >1 month between the ultrasound examination and EGFR testing; (ii) the presence of concurrent primary malignancies and (iii) unsatisfactory US, CEUS and TIC images. The final study population included 75 individuals (30 females, 45 males) with an average age of 64.08±11.01 years. All 75 enrolled patients including 47 patients tested by our internal pathology laboratory and 28 cases tested by external laboratories holding equivalent accreditation were tested for EGFR mutations using Amplification refractory mutation system polymerase chain reaction (ARMS-PCR) method. The commercial ADx-EGFR Mutation Detection Kit (AmoyDx) was utilized on the ABI 7500 Real-Time PCR system (Applied Biosystems). This assay is designed to detect 29 somatic mutations in the EGFR gene, including exon 19 deletions, the T790M mutation, and mutations in exons 18, 20, and 21 (such as L858R and L861Q). The patient selection flowchart was illustrated in **Figure 1**.

### Ethical approval

We confirmed that this study adherences to the Declaration of Helsinki. This study was approved by our local ethics committee, and written informed consent was waved because of retrospective study.

### CEUS examination methods

The contrast agent was SonoVue (Bracco SpA, Italy). The lyophilized powder was reconstituted with 5 mL of saline to produce a uniform microbubble suspension containing 8 µL/mL of sulfur hexafluoride encapsulated in a phospholipid shell. A 2.4 mL bolus of the suspension was injected into the antecubital vein over 2–3 s. CEUS images were acquired using the Logiq E9 ultrasound system. The frequency and depth settings used for CEUS were identical to those of conventional US. The mechanical index was adjusted to 0.11–0.13. The timer was activated simultaneously with contrast injection. Lesions were assessed using contrast-enhanced harmonic US with continuous recording for ≥ 180 seconds. Dynamic images were saved to the internal hard drive of the ultrasound system.

Enhancement process were classified as arterial and parenchymal phases, beginning at contrast administration. The arterial phase was defined as the interval from the initial appearance of the contrast agent in the lesion to the point of peak enhancement. Time-intensity curves were generated using the built-in postprocessing software of the Logiq E9 ultrasound equipment.

### Evaluation of US, CEUS, and TIC

Two radiologists (W.Y. and J.B.), each with 5–10 years of experience in lung ultrasound and contrast-enhanced ultrasound, independently evaluated the US and CEUS images. They jointly reviewed the imaging data and formulated consensus reports. Both radiologists were blinded to the patients’ EGFR mutation status.

In the US assessment, lesion boundaries were classified as well defined or poor defined. Lesion shapes were categorized as suborbicular, irregular, or wedge-shaped. Using surrounding atelectasis normal lung tissue as a reference, echogenicity was defined as hypoechoic or isoechoic. An air bronchogram was defined as a tubular or branching air-filled structure within the tumor, resulting from ultrasound reflections of residual air-filled bronchi with posterior comet-tail artifacts. Pleural retraction was described as inward pleural displacement toward the tumor, attributed to fibrotic contraction within tumor tissue.

In CEUS evaluation, enhancement patterns during the arterial phase were classified as: (i) centripetal perfusion, where contrast agent entered from the periphery toward the center; (ii) eccentric perfusion, where perfusion began on one side of the lesion and extended across; and (iii) disorganized enhancement, where perfusion occurred randomly throughout the lesion. Necrosis types were defined as: (i) type A, no necrosis; (ii) type B, single or multiple necrotic foci. Hydrothorax was recorded as present or absent. Homogeneity was classified as present or absent based on uniform echo intensity within the lesion, excluding necrotic areas at peak contrast enhancement. Enhancement intensity, relative to adjacent normal lung tissue, was classified as hyperenhancement or hypoenhancement.

The CEUS video was analyzed frame by frame to generate the TIC. The enhancement arrival time (AT) was defined as the interval from contrast injection to the initial enhancement of the lesion. The rise time (RT) was defined as the time from initial enhancement to peak enhancement. The time to peak (TTP) was calculated as the sum of AT and RT. The peak intensity (PI) was defined as the echo intensity at peak enhancement. The wash-in area under the curve (Wi-AUC) represented the area under the TIC during the wash-in phase of contrast enhancement.

### Statistical analysis and model development

All statistical analyses were performed using R software (version 4.4.3). The 75 patients were stratified into EGFR mutant and EGFR wild-type groups based on mutation status. Categorical variables were described as frequencies and percentages, while continuous variables were reported as mean±standard deviation (SD) or median with range. Categorical variables were compared using the χ² test or Fisher’s exact test. Continuous variables were analyzed using Student’s t-test or the Mann–Whitney U-test, as appropriate. A p-value of <0.05 was considered statistically significant.

Variables showing statistical significance in clinical characteristics, US features, CEUS findings, and TIC parameters were further analyzed using univariate and stepwise multivariate logistic regression. Odds ratios (ORs) were calculated for all variables included in multivariate analysis. Two predictive models and their corresponding nomograms were constructed based on the multivariate logistic regression results. The discriminative performance of models was evaluated through Receiver operating characteristic (ROC). The area under the curve (AUC) was calculated, and the optimal cutoff value was determined using the Youden index. Calibration curves were used to assess model calibration, and decision curve analysis (DCA) evaluated clinical utility.

## Results

### Clinical characteristics

A total of 75 patients were enrolled in this study. Patients were stratified into an EGFR wild-type group (n = 57) and an EGFR-mutant group (n = 18) based on EGFR mutation status. The clinical characteristics and corresponding statistical comparisons between the two groups were presented in **Table 1**. Statistically significant differences between groups were observed in sex (*p* = 0.001) and smoking status (*p* < 0.001). Meanwhile, no significant differences were observed in age (p = 0.816), TNM stage (p = 0.312), histological subtype (p = 0.133), tumor location (p = 0.861), and tumor size (p = 0.664) between EGFR+ and EGFR- groups.

**Table 1:** Comparison of clinical characteristics of patients with or without EGFR mutation.

Variable	EGFR- (N=57)	EGFR+ (N=18)	*p*
Age, *Mean ± SD*	63.91 ± 10.13	64.61 ± 13.78	0.816
Sex, *n (%)*			0.001**
Female	17 (29.82%)	13 (72.22%)	
Male	40 (70.18%)	5 (27.78%)	
TNM Stage, *n (%)*			0.312
Available	60 (80.00%)		
I-III	13	2	
IV	31	14	
NA	15 (20.00%)		
Smoking status, *n (%)*			<0.001***
Former	41 (71.93%)	5 (27.78%)	
Never	16 (28.07%)	13 (72.22%)	
Histological subtype, *n (%)*			0.133
Adenocarcinomas	40 (70.18%)	16 (88.89%)	
Squamous cell carcinomas	17 (29.82%)	2 (11.11%)	
Tumor location, *n (%)*			0.861
Left	24 (42.11%)	8 (44.44%)	
Right	33 (57.89%)	10 (55.56%)	
Size, *Median (Q1-Q3)*	5.72 (4.72-7.55)	6.02 (3.11-8.25)	0.664

EGFR, epidermal growth factor receptor; TNM, tumor node metastasis; NA, not applicable.

**p < 0.01, ***p < 0.001. Bold values indicate p < 0.05.

### Development of models

The US features, CEUS characteristics, and TIC parameters were summarized in **Table 2**. Significant differences were identified in US features, including lesion boundaries (p = 0.017) and air bronchogram (p = 0.012). In CEUS, enhancement pattern (p = 0.034), homogeneity (p < 0.001), enhancement intensity (p = 0.026), and internal necrosis (p = 0.011) differed significantly between the two groups. Additionally, there was significant difference in RT on TIC analysis (p = 0.015).

**Table 2:** Comparsion of US, CEUS and TIC features of lung lesions with or without EGFR mutation.

Variable	EGFR- (N=57)	EGFR+ (N=18)	*p*
US						
Boundaries, *n (%)*			0.017*
Well defined	26 (45.61%)	14 (77.78%)	
Poor defined	31 (54.39%)	4 (22.22%)	
Shape, *n (%)*			0.816
Suborbicular	27 (47.37%)	10 (55.56%)	
Irregular	27 (47.37%)	7 (38.89%)	
Wedge-shaped	3 (5.26%)	1 (5.56%)	
Echo, *n (%)*			0.567
Hypoecho	55 (96.49%)	17 (94.44%)	
Iso echo	2 (3.51%)	1 (5.56%)	
Air bronchogram, *n (%)*			0.012*
Yes	19 (33.33%)	12 (66.67%)	
No	38 (66.67%)	6 (33.33%)	
Pleural retraction, *n (%)*			0.982
Yes	22 (38.60%)	7 (38.89%)	
No	35 (61.41%)	11 (61.11%)	
CEUS						
Enhancement pattern, *n (%)*			0.034*
Centripetal	37 (64.91%)	12 (66.67%)	
Eccentric	5 (8.77%)	5 (27.78%)	
Disorganized	15 (26.32%)	1 (5.56%)	
Homogeneity, *n (%)*			<0.001***
Yes	14 (24.56%)	13 (72.22%)	
No	43 (75.44%)	5 (27.78%)	
Enhancement intensity, *n (%)*			0.026*
Hyperenhancement	24 (42.11%)	13 (72.22%)	
Hypoenhancement	33 (57.89%)	5 (27.78%)	
CEUS washout type, *n (%)*			0.499
Early washout	48 (84.21%)	14 (77.78%)	
Late washout	9 (15.79%)	4 (22.22%)	
CEUS washout degree, *n (%)*			0.251
Complete	36 (63.16%)	14 (77.78%)	
Incomplete	21 (36.84%)	4 (22.22%)	
Necrosis, *n (%)*			0.011*
Yes	41 (71.93%)	7 (38.89%)	
No	16 (28.07%)	11 (61.11%)	
Hydrothorax, *n (%)*			0.536
Yes	12 (21.05%)	5 (27.78%)	
No	45 (78.95%)	13 (72.22%)	
TIC						
AT, Mean ± SD	8.96 ± 3.18	8.82 ± 1.85	0.816
RT, Median (IQR)	14.00 (12.00-17.00)	15.46 (15.46-18.75)	0.015*
TTP, Mean ± SD	23.36 ± 5.77	25.47 ± 4.78	0.164
PI, Mean ± SD	20.59 ± 4.19	19.17 ± 4.17	0.216
WiAUC, Mean ± SD	207.80 ± 69.46	235.20 ± 42.98	0.119

TIC, time-intensity curve; AT, Arrive time; RT, Rise time; TTP, Time to peak; PI, Peak intensity; WiAUC, Wash in area under curve.

*p < 0.05, ***p < 0.001. Bold values indicate p < 0.05.

**Figure 2:**
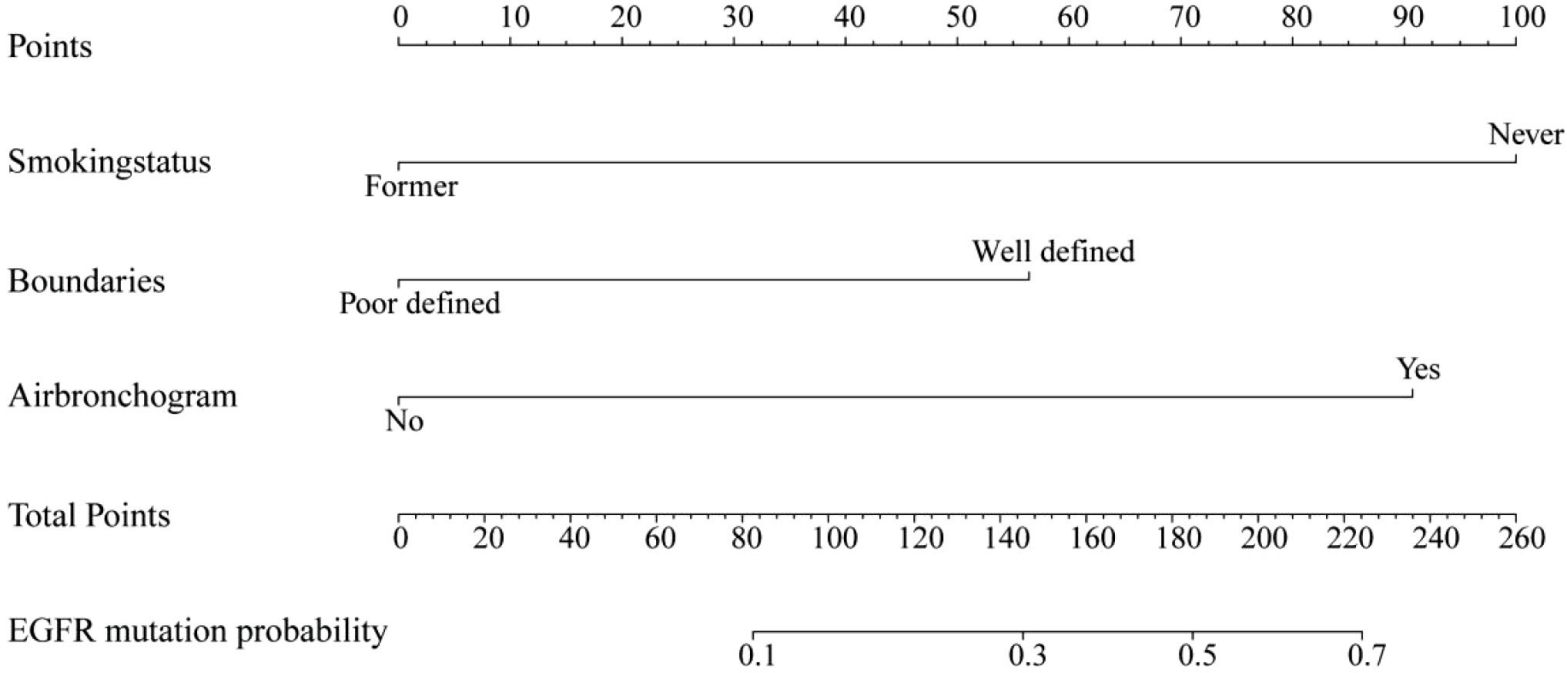
Nomogram for C+US model. C, Clinical characteristics; US, ultrasound; EGFR, Epidermal Growth Factor Receptor.

The results of univariate logistic regression for all statistically significant parameters were displayed in **Table 3**. The clinical characteristics (sex, smoking status) and US features (boundaries, air bronchogram) were entered into a stepwise multivariate logistic regression. Smoking status (p = 0.003), lesion boundaries (p = 0.090) and air bronchogram (p = 0.007) were identified as independent predictors. A combined C + US model was constructed using these three variables (**Table 4**), and its corresponding nomogram was illustrated in **Figure 2**.

**Table 3:** Univariate logistic regression analysis of clinical, US and CEUS features.

Variable	Univariate analysis		
	Beta	OR [95%CI]	*p*
Sex			
Female	1.81	6.12 [1.98-21.64]	0.003
Male		1 [Reference]	
Smoking status			
Former	-1.90	0.15 [0.042-0.47]	0.002
Never		1 [Reference]	
Boundaries			
Well defined	1.43	4.17 [1.31-16.15]	0.023
Poor defined		1 [Reference]	
Air bronchogram			
Yes	1.39	4.00 [1.34-13.08]	0.016
No		1 [Reference]	
Enhancement pattern			
Centripetal	1.58	4.86 [0.84-92.55]	0.145
Eccentric	2.71	15.00 [1.86-327.36]	0.025
Disorganized		1 [Reference]	
Homogeneity			
Yes	2.08	7.99 [2.54-28.75]	<0.001
No		1 [Reference]	
Enhancement intensity			
Hypoenhancement	-1.27	0.28 [0.08-0.85]	0.031
Hyperenhancement		1 [Reference]	
Necrosis			
Yes	-1.39	0.25 [0.08-0.74]	0.014
No		1 [Reference]	
RT	0.13	1.14[1.01-1.32]	0.048

C, Clinical characteristics; US, ultrasound; CEUS, contrast enhanced ultrasound; RT, Rise time.

**Table 4:** Stepwise multivariate logistic regression analysis for C+US model.

Variable	Beta	S.E	Z	OR [95%CI]	P
Smoking status					
Former	-2.15	0.72	-2.99	0.12 [0.02-0.44]	0.003
Never				1 [Reference]	
Boundaries					
Well defined	1.21	0.72	1.69	3.36[0.88-15.36]	0.090
Poor defined				1 [Reference]	
Air bronchogram					
Yes	1.95	0.72	2.72	7.02[1.88-32.84]	0.007
No				1 [Reference]	

C, Clinical characteristics; US, ultrasound.

**Figure 3:**
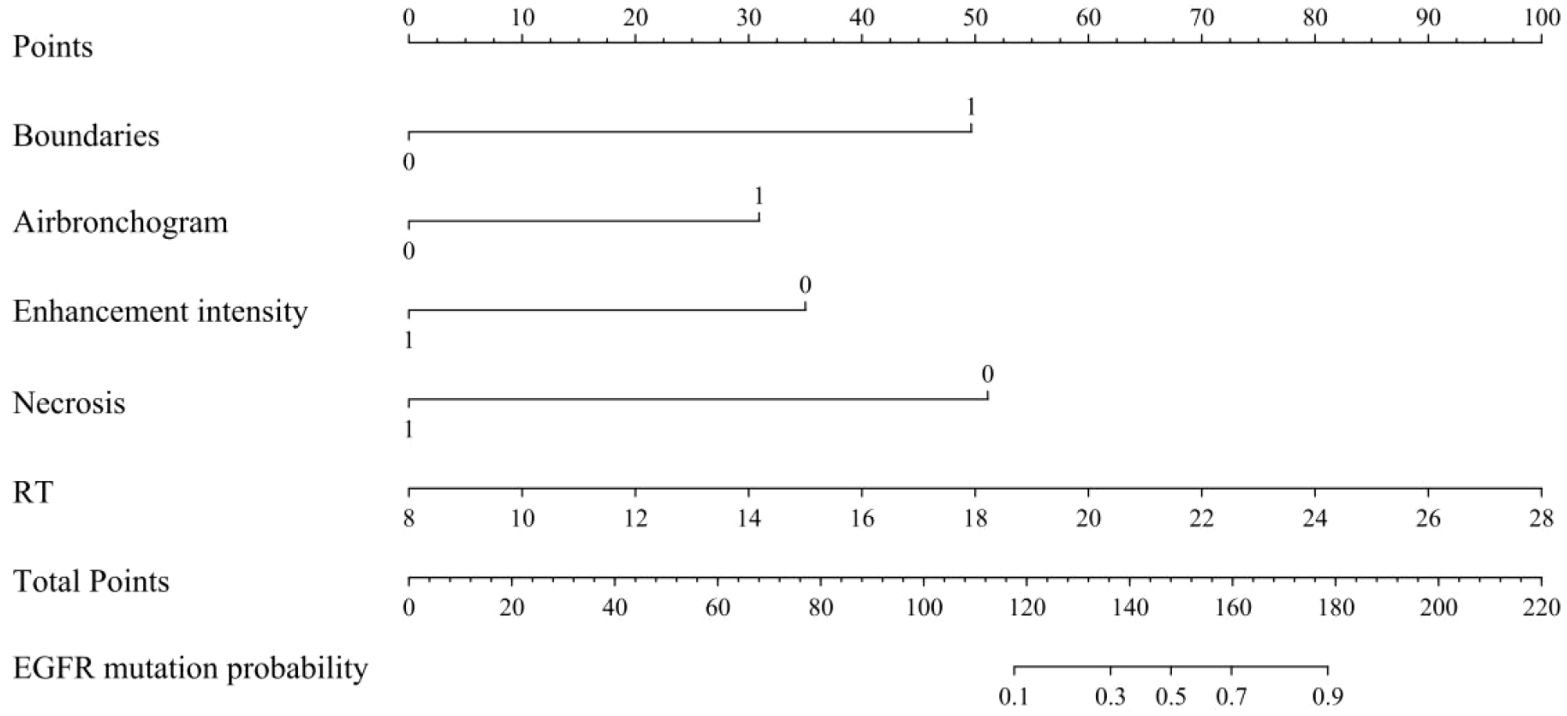
Nomogram for the FULL model. RT, rise time; EGFR, Epidermal Growth Factor Receptor.

**Figure 4:**
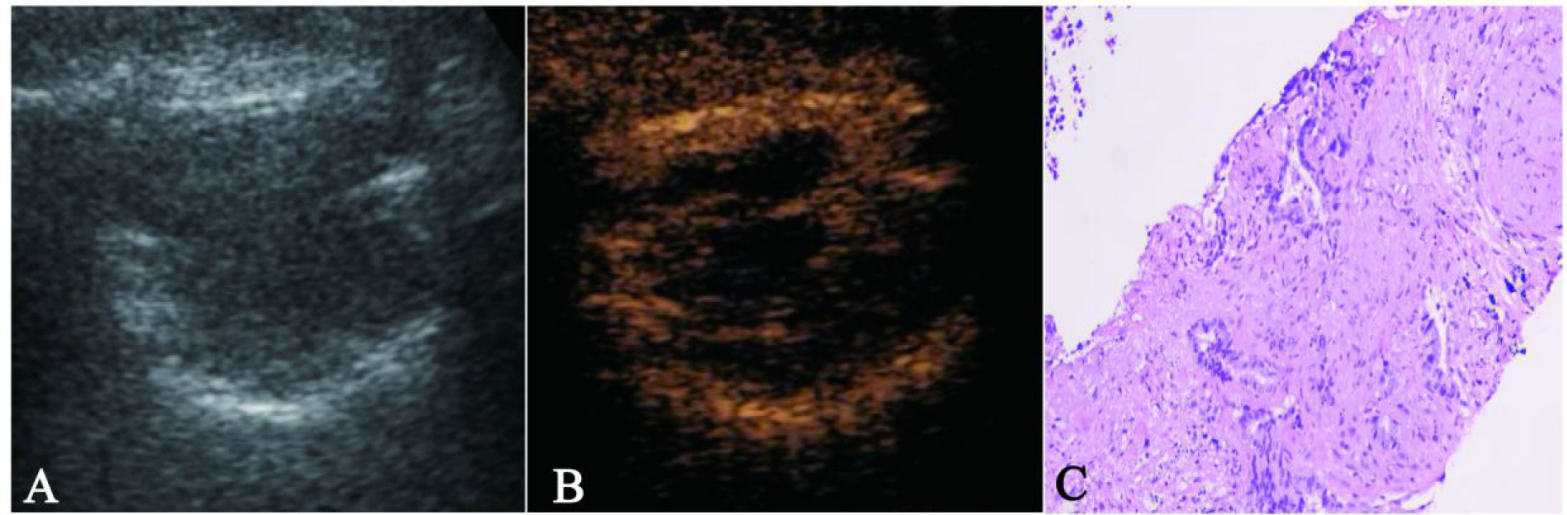
The figure 4A–4C showed a case of 70-year-old male NSCLC with EGFR-wild type. **(A)** The gray-scale ultrasound shown a hypo-echoic lesion with poor defined boundary. **(B)** On the contrast-enhanced ultrasound, the lesion exhibited hypo-enhancement with multiple necrotic focis. **(C)** The pathological image confirmed the lesion as a NSCLC.

**Figure 5:**
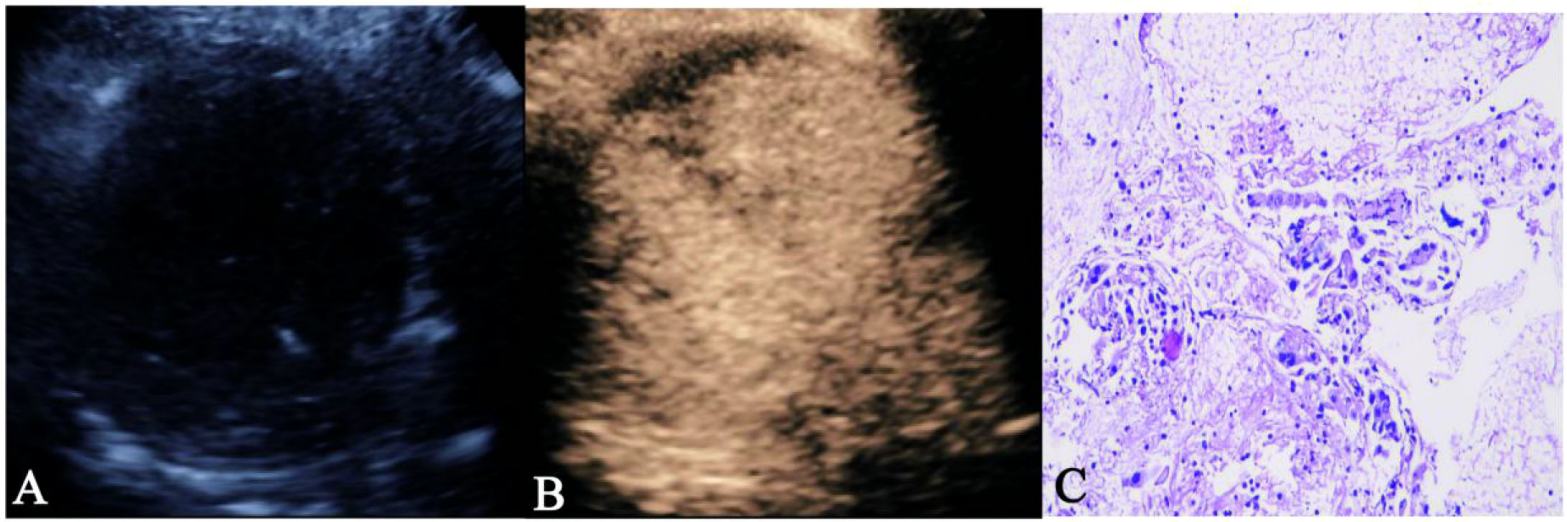
The 5A–C showed a case of 50-year-old female NSCLC with EGFR-mutant. **(A)** The gray-scale ultrasound shown a hypo-echoic lesion with well defined boundary and air bronchograms. **(B)** On the contrast-enhanced ultrasound, the lesion exhibited homogeneous hyperenhancement. **(C)** The pathological image confirmed the lesion as a NSCLC.

Subsequently, clinical characteristics (sex, smoking status), US features (boundaries, air bronchogram), CEUS characteristics (homogeneity, enhancement intensity, necrosis), and the TIC parameter RT were analyzed via stepwise multivariate logistic regression. Lesion boundaries (p = 0.002), air bronchogram (p = 0.013), enhancement intensity (p = 0.020), internal necrosis (p = 0.002), and RT (p = 0.008) were identified as independent predictors for EGFR status. Based on these five variables, the FULL model was developed (**Table 5**), with a corresponding nomogram shown in **Figure 3**. **Figures 4A–C**, **5A–C** demonstrated ultrasound images and pathological image of EGFR-wild type and EGFR-mutant lesions, respectively.

**Table 5:** Stepwise multivariate logistic regression analysis for FULL model.

Variable	Beta	S.E	Z	OR [95%CI]	P
Boundaries					
Well defined	3.58	1.18	3.03	35.99 [4.98-608.62]	0.002
Poor defined				1 [Reference]	
Air bronchogram					
Yes	2.23	0.90	2.48	9.31[1.83-67.96]	0.013
No				1 [Reference]	
Enhancement intensity					
Hypoenhancement	-2.53	1.09	-2.33	0.07[0.01-0.52]	0.020
Hyperenhancement				1 [Reference]	
Necrosis					
Yes	-3.69	1.17	-3.14	0.03 [0.001-0.18]	0.002
No				1 [Reference]	
RT	0.36	0.14	2.64	1.43 [1.13-1.97]	0.008

RT, Rise time.

### Performance and validation of two models

**Figure 6:**
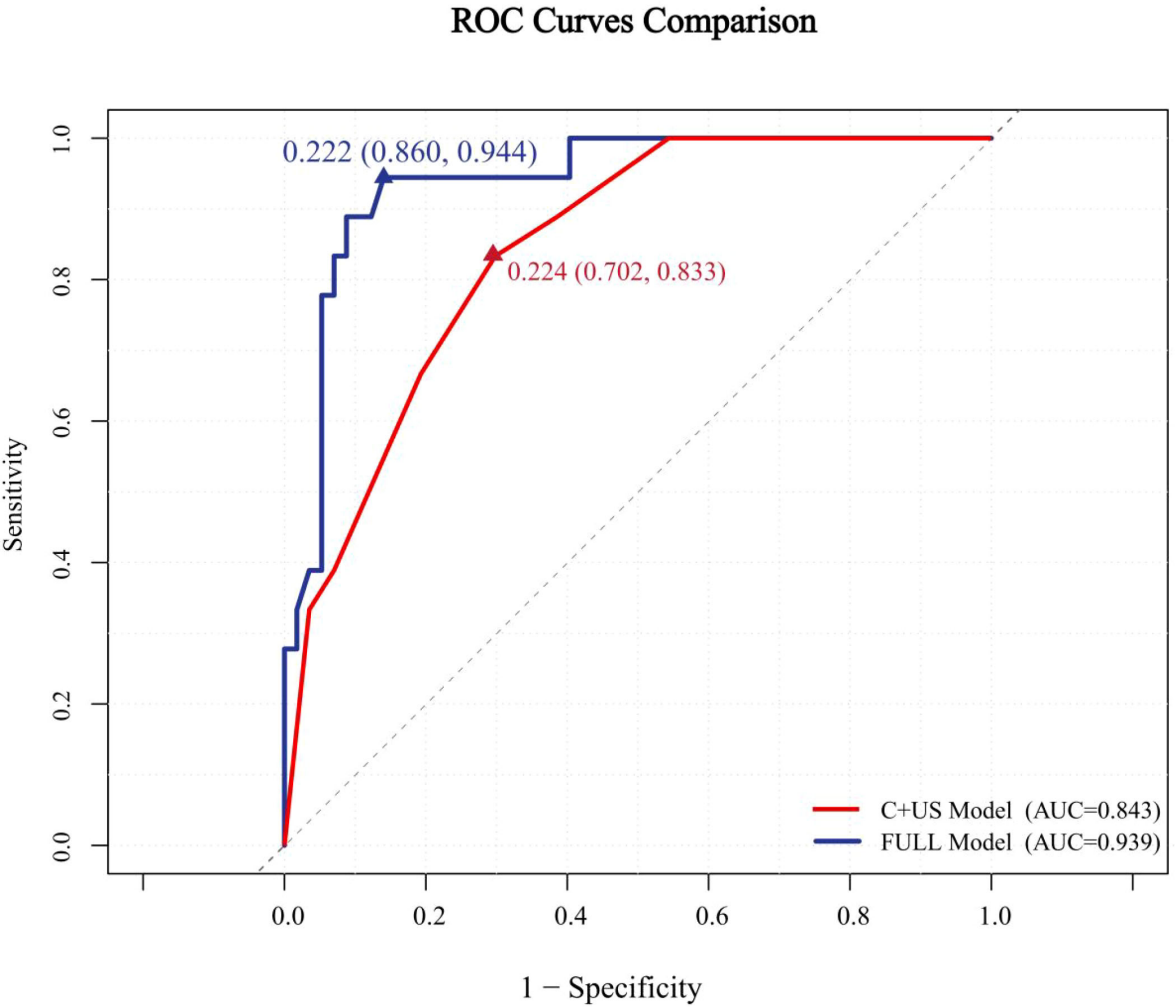
Receiver operating characteristic (ROC) curves of the two models with AUC of 0.843 and 0.939. C, Clinical characteristics; US, ultrasound.

**Figure 7:**
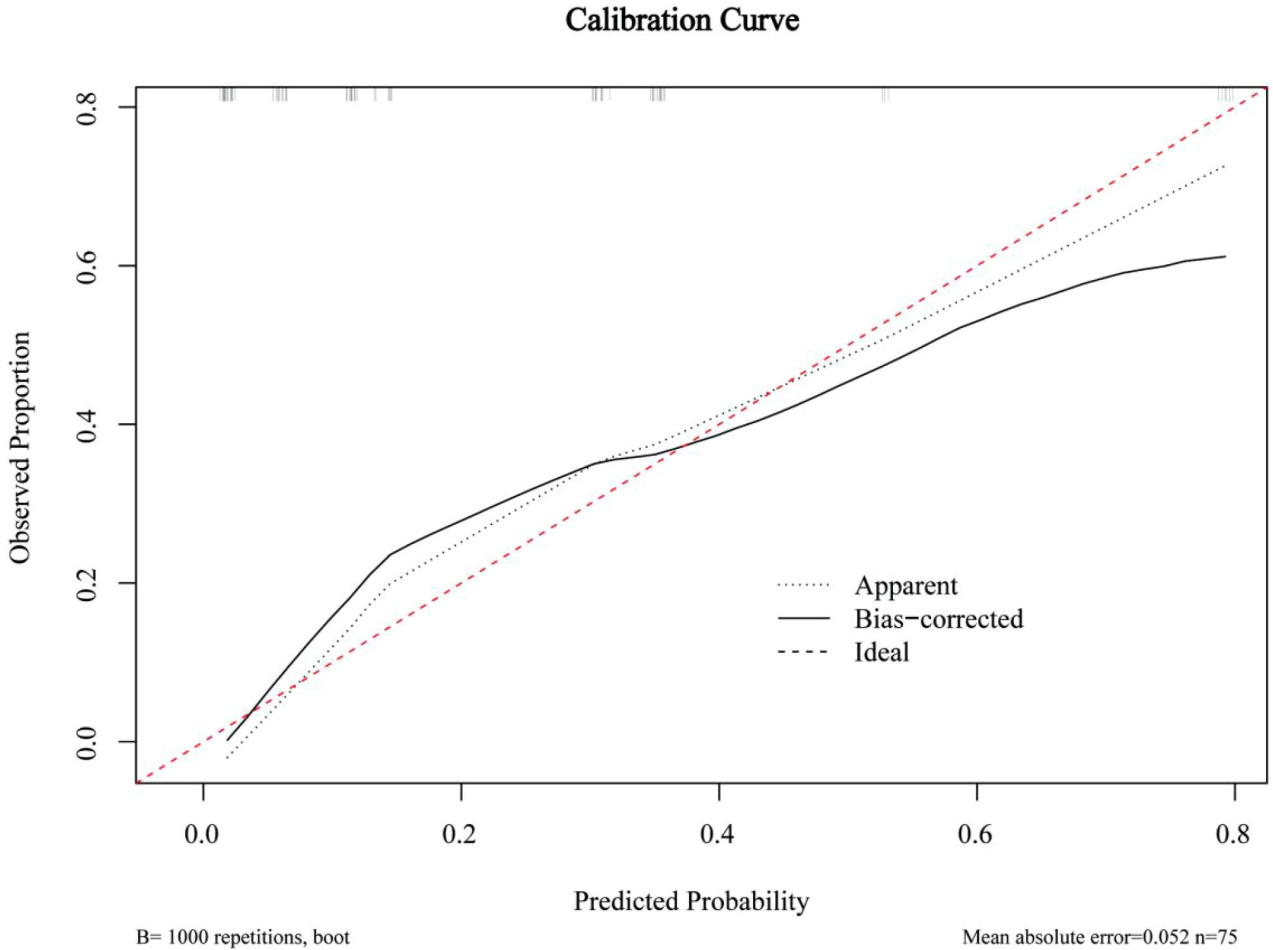
Calibration curves of C+US model. C, Clinical characteristics; US, ultrasound.

**Figure 8:**
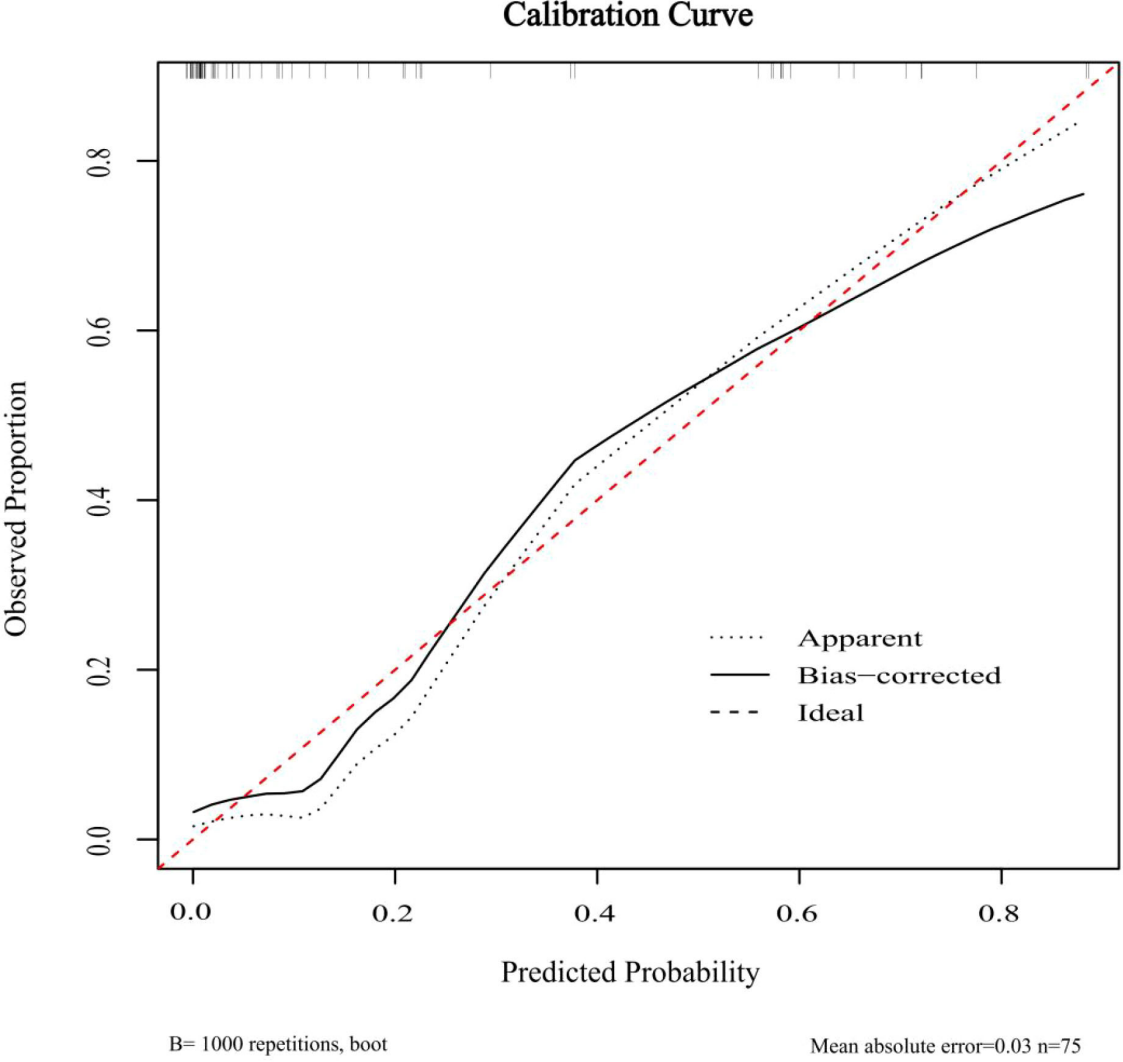
Calibration curves of FULL model. C, Clinical characteristics; US, ultrasound; CEUS, contrast enhanced ultrasound; NSCLC, non-small cell lung cancer; TIC, time-intensity curve.

**Figure 9:**
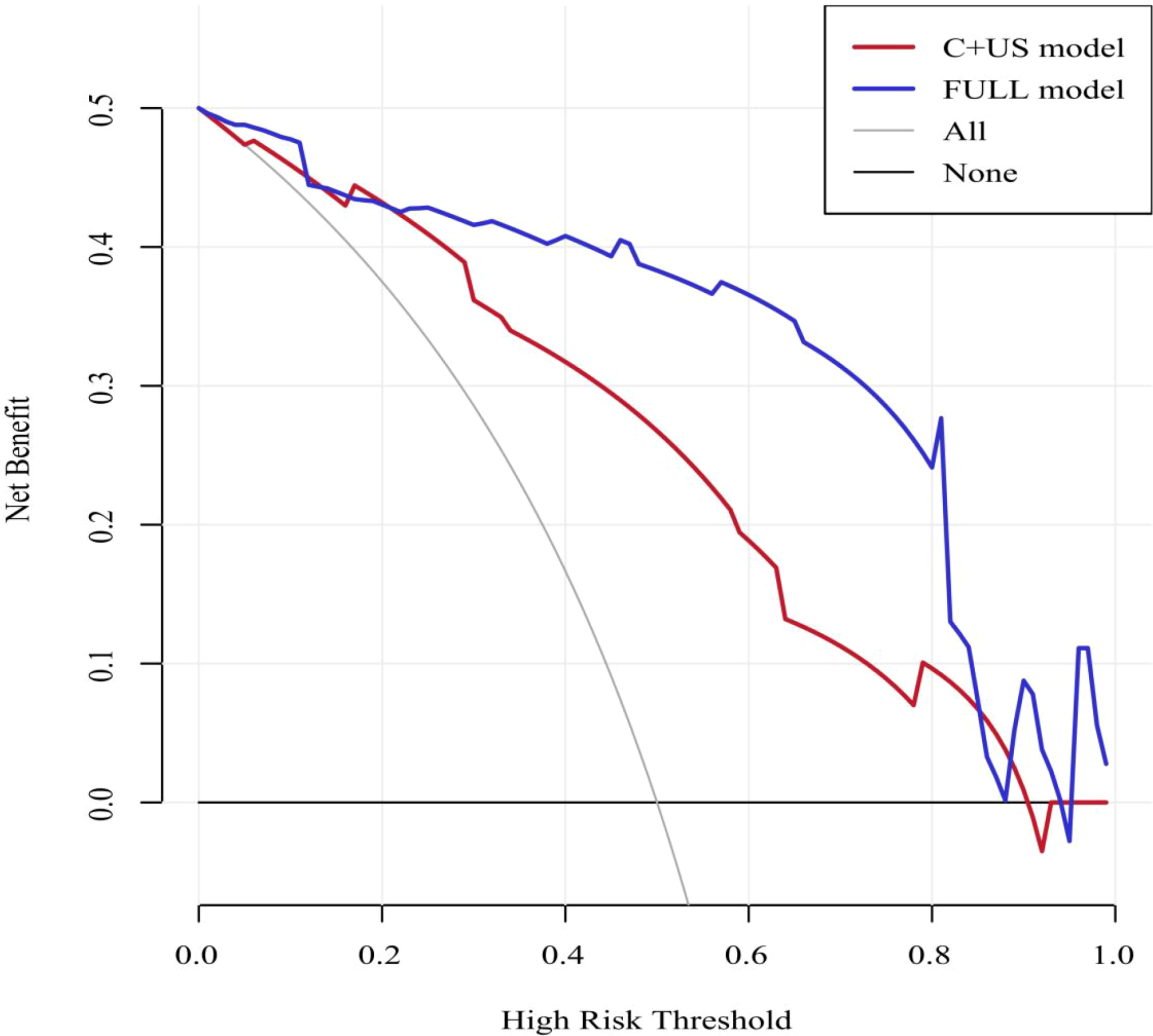
Decision curve analysis showing the net clinical benefit of the two models. C, Clinical characteristics; US, ultrasound; NSCLC, non-small cell lung cancer.

The ROC curves for both predictive models were presented in **Figure 6**. The FULL model demonstrated superior diagnostic performance compared to the C + US model (AUC: 0.939 vs. 0.843, p < 0.05). The predictive metrics of both models, including specificity, sensitivity, negative predictive value, positive predictive value, and accuracy were summarized in **Table 6**. Calibration curves (**Figures 7**, **8**) demonstrated high concordance of predictions with observations in both models. DCA further confirmed the clinical utility of both models. As shown in **Figure 9**, the FULL model provided greater net clinical benefit than the C + US model across most threshold probabilities. The FULL model demonstrated a substantial improvement in predictive performance compared to the C+US model, attributable to the integration of CEUS features and TIC parameters.

**Table 6:** Predictive performance of two models.

Model	AUC	Sensitivity (%)	Specificity (%)	Positive predictive value	Negative predictive value	Accuracy
C+US	0.843	83.3	70.2	73.7	80.8	76.8
FULL	0.939	94.4	86.0	87.1	93.9	90.2

C, Clinical characteristics; US, ultrasound.

## Discussion

Since targeted therapy provides meaningful survival benefits and quality-of-life improvements in advanced lung cancer, accurate detection of EGFR status before treatment is crucial throughout clinical management.

Multiple studies support the predictive value of radiomic features for EGFR genotype in NSCLC (10, 11, 15, 16). Several studies have suggested that female individuals and never-smokers are more likely to harbor EGFR mutations (17–19). Additionally, prior research using computed tomography (CT) and PET/CT imaging has demonstrated that imaging features such as ground-glass opacity, convergence of surrounding vessels, pleural retraction, and adjacent invasion are correlated with EGFR mutation status (20–22). Compared to CT, US, particularly CEUS, offers real-time monitoring, absence of radiation exposure, accessibility, and cost-effectiveness, making it a viable supplemental imaging tool for the differential diagnosis of subpleural pulmonary lesions. Existing studies have confirmed the high sensitivity of US and CEUS in diagnosing pulmonary and pleural lesions and in distinguishing benign from malignant pathologies (13). To our knowledge, this is the first study to predict EGFR mutation status in NSCLC with ultrasound. Our findings demonstrated that US enables noninvasive, radiation-free prediction of EGFR status with robust diagnostic performance.

In our study, univariate analysis revealed a predominance of female and non-smoking patients in the EGFR-mutant group, consistent with previous studies (10, 11). Prior research has demonstrated that adenocarcinoma is more prone to EGFR mutations. Although our cohort included squamous cell carcinoma cases, no significant association between histologic type and EGFR status was observed, possibly due to the relatively small sample size (23). In the US results, well defined boundaries and air bronchograms were exhibited predominantly in the EGFR-mutant group. This imaging phenotype may relate to the lepidic and expansile growth patterns linked to EGFR mutations. Preserved bronchial structure and air-filled spaces enable bronchogram formation, while the expansile growth pattern likely contributes to well-defined tumor boundaries (20). Regarding CEUS findings, lesions with EGFR mutations were more likely to exhibit homogeneous hyperenhancement and lack necrosis. This imaging phenomenon may reflect EGFR downstream signaling. EGFR mutations activate PI3K–AKT–mTOR pathways, upregulating hypoxia-inducible factor-1 (HIF-1), which promotes vascular endothelial growth factor (VEGF) production. VEGF induces neovascularization by influencing endothelial cells in existing vessels and promoting their migration. The development of abnormal vasculature promotes tumor growth and metastasis. Enhanced vascularization may explain the observed homogeneous hyperenhancement and reduced necrosis (24, 25).

Our study had several limitations. First, the relatively small sample size and limited number of EGFR‐mutant events may compromise the stability of the multivariable model and restrict its generalizability to broader populations. Therefore, the model is exploratory and we will further confirm the results with large samples in multiple centers in the next study. Second, the long study period (2012–2024) introduces the potential of temporal bias, as clinical management, EGFR testing techniques, and ultrasound/CEUS equipment and protocols may have evolved over time. Because the EGFR testing became popular for lung cancer patients in the recent years, we believe we can collect more data during short term period in the future. Third, beyond EGFR, other driver mutations are also relevant to targeted therapy, which need further investigate in the future.

## Conclusions

The models developed in this study enable patients who are unable to undergo invasive procedures to predict EGFR mutation status noninvasively. These findings provided an ultrasound-based diagnostic reference to support clinician decision-making and personalized treatment planning.

## Data Availability

The original contributions presented in the study are included in the article/supplementary material. Further inquiries can be directed to the corresponding authors.
